# Duodenal Ulcer

**Published:** 1914-04

**Authors:** 


DUODENAL ULCER. Ewald has encountered in the last three and a half years 532 cases of gastric and eighty-two of duodenal ulcer. He found blood in the stools the most constant sign of the latter: it was present in every case with one exception, but not always at the first examination. He declares that about 50 per cent. of all duodenal ulcers are amenable to medical treatment. On the other hand, operative treatment has its drawbacks; in one case the patient had a severe hemorrhage from the duodenal ulcer two months after it had been treated by gastro-enterostomy. He advised an operation only in eighteen of his eighty-two cases.

